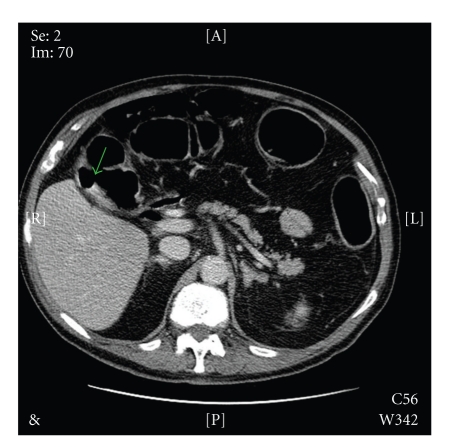# Gallstone Ileus of the Sigmoid Colon: An Unusual Cause of Large-Bowel Obstruction

**DOI:** 10.1155/2010/153740

**Published:** 2010-05-31

**Authors:** Nadir Osman, Daren Subar, Mong-Yang Loh, Andrzej Goscimski

**Affiliations:** ^1^Department of Surgery, Stepping Hill Hospital, Stockport, Manchester SK2 7JE, UK; ^2^Department of Radiology, Stepping Hill Hospital, Stockport, Manchester SK2 7JE, UK

## Abstract

Gallstone ileus of the colon is an exceedingly rare cause of large-bowel obstruction. It is usually the result of fistula formation between the gallbladder and large bowel facilitating entry of the stone into gastrointestinal tract. Contrast enhanced abdominal computed tomography is an important diagnostic aid. Surgical management is the treatment of choice to prevent the disastrous complications of large-bowel obstruction. We describe the case of a 92-year-old man who presented with symptoms and signs of large-bowel obstruction. Radiological investigation showed a large gallstone impacted in the sigmoid colon. Open enterolithotomy was undertaken relieving the obstruction and the patient made a full recovery.

## 1. Introduction

Colonic gallstone ileus is an exceedingly rare cause of mechanical large-bowel obstruction. It occurs most commonly due to the passage of a solitary large stone or several smaller stones through a cholecystocolonic fistula into the colon. It is a disease of high morbidity and mortality due to the diagnostic challenge, late presentation, advanced patient age, and comorbid states. We report a case of gallstone ileus of the sigmoid colon and review the available literature.

## 2. Case History

A 92-year-old male was admitted to hospital with a 3-day history of intermittent colicky lower abdominal pain associated with absolute constipation and abdominal distension. Clinical examination revealed a distended abdomen with Left iliac fossa tenderness but no overt peritonism. 

He had an episode of acute cholecystitis in the previous year. A CT scan at that time demonstrated a 3.8 cm stone in the gallbladder. No subsequent intervention was planned due to his cardiorespiratory comorbidities.

**Figure 1:**
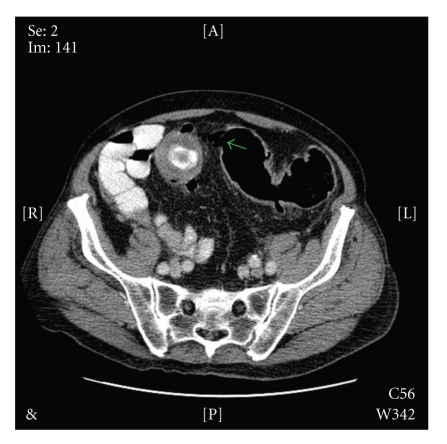


Biochemical and haematological investigations revealed a CRP of 87.6 mg/L and a white cell count of 21.2 × 10^9^/L. Liver function tests and serum amylase were entirely normal. Initial abdominal X-rays showed gaseous distension of the large bowel with no evidence of pneumobilia. A subsequent contrast-enhanced CT scan of the abdomen demonstrated a 3.8 cm opacity impacted in the midsigmoid colon (Figure 1) associated with secondary inflammatory change and multiple diverticulae. A fistulous connection between a collapsed empty gallbladder and the hepatic flexure of the colon was also identified (Figure 2).

At laparotomy an enterolithotomy was performed. The cholecystocolonic fistula was identified but was not repaired in order to minimise the operative time and hence the risk of perioperative complications. The diverticulous segment of large bowel at the point of impaction was also not resected for the same reason. The patient was eventually discharged to the rehabilitation ward 4 weeks after admission.

## 3. Discussion

Gallstone ileus (GSI) of the colon is an extremely rare cause of large-bowel obstruction. It has been shown to account for 2% to 8% of all cases of GSI [1, 2]. It is a disease of the elderly with considerable morbidity and mortality.

The mechanism of obstruction usually results from a large gallstone greater than 2 cm in diameter entering the colon through a cholecystocolonic fistula. The fistulous connection usually forms after a preceding episode acute cholecystitis leads to inflammation and adhesions forming between the gallbladder and colon, usually at the hepatic flexure. The gallstone impacts distally and causes a mechanical obstruction. The point of impaction is usually a pathological narrowing of the colon which may be a result of diverticular disease [3, 4] or prior pelvic irradiation [5]. 

The typical presentation is that of large-bowel obstruction with abdominal pain and distension being the main features. However, rarer presentations such as diarrhoea or ascending cholangitis can occur [6]. As in GSI of the small bowel, the classic X-ray triad of bowel obstruction, pneumobilia, and ectopic gallstones [7] is sometimes but not always seen. Contrast-enhanced computed tomography allows better visualisation of the point of obstruction and site of fistula [8, 9].

About 7% of GSI have been shown to settle with conservative management. However, in the majority of cases operative management is required. In the main part operative intervention for colonic GSI is the same as small bowel GSI. The definitive management involves enterolithotomy, cholecystectomy, and fistula closure at one operation [10]. This allows relief of the obstruction as well as preventing further stone formation and episodes of cholangitis. For the higher-risk patient such as in our report a single stage enterolithotomy alone may be more appropriate. During either operation the entire length of the small and large bowel should be examined for additional occult stones. Management by colonoscopy and extraction has been successful in few cases [11, 12]. However when the stone is relatively large and the lumen is relatively small, it is less likely to be successful [13].

In conclusion GSI of the colon is a rare but important cause of large-bowel obstruction. The diagnosis may be very difficult given the lack of distinguishing clinical features but should be considered if there has been a history of gallstone disease. If plain abdominal films do not reveal the diagnosis, computed tomography will allow visualisation of the fistula and level of obstruction. Treatment depends on the fitness of the patient but should ideally involve relief of the obstruction, cholecystectomy, and repair of the fistula.

## Figures and Tables

**Figure 2:**